# Seasonal Variation in Essential Minerals, Trace Elements, and Potentially Toxic Elements in Donkey Milk from Banat and Balkan Breeds in the Zasavica Nature Reserve

**DOI:** 10.3390/ani15060791

**Published:** 2025-03-11

**Authors:** Dragana Ljubojević Pelić, Nenad Popov, Ema Gardić, Suzana Vidaković Knežević, Marina Žekić, Vladimir Gajdov, Milica Živkov Baloš

**Affiliations:** Scientific Veterinary Institute “Novi Sad”, 21000 Novi Sad, Serbia; nenad.p@niv.ns.ac.rs (N.P.); ema.g@niv.ns.ac.rs (E.G.); suzana@niv.ns.ac.rs (S.V.K.); marina@niv.ns.ac.rs (M.Ž.); vladimir.g@niv.ns.ac.rs (V.G.)

**Keywords:** autochthonous donkey breeds, food safety, heavy metals, minerals

## Abstract

Donkey milk is highly valued for its unique nutritional characteristics and hypoallergenic properties. However, limited data exist on its mineral composition and potential contaminants. This study aimed to comprehensively analyze the concentration of selected essential and toxic elements from Banat and Balkan donkey breeds reared under extensive organic conditions (free-range) across different seasons. The focus was on determining the concentrations of essential minerals and trace elements (Ca, P, Na, K, Mg, Zn, Cu, Se), and potentially toxic elements (As, Pb, Hg, Cd). Samples were collected monthly over one year, with nine pooled samples per season. Mineral elements were determined using a validated ICP-MS method. The mineral content ranges (mg/kg) were Ca (588.9–744.4), P (355.6–533.3), Mg (71.1–84.4), K (444.4–711.1), Na (355.6–444.4), with trace elements (mg/kg) including Zn (2.06–2.21), Cu (0.31–0.68), and Se (0.02–0.05). Potentially toxic elements were within safe limits, with the exception that Pb levels exceeded permissible limits in two samples. Statistically significant seasonal variations were observed for Ca, P, K, As, and Cu. Donkey milk from both breeds was rich in minerals and low in contaminants. This research highlights the need for further studies on the nutritional and safety aspects of donkey milk.

## Simple Summary

Donkey milk is known for its unique nutritional benefits and hypoallergenic properties, but there is limited information on its mineral content and potential contaminants. This study aimed to explore the nutritional quality and safety of milk from Banat and Balkan donkey breeds raised in the Zasavica Natural Reserve in Serbia, over different seasons. The researchers measured important minerals such as calcium, phosphorus, and zinc, as well as harmful substances like lead and cadmium, which can be toxic. The results showed that donkey milk contains high levels of essential minerals, with some seasonal variations, and was generally free from harmful contaminants. However, the study found that lead levels in spring were higher than the permissible limits in a few samples. Overall, the findings suggest that donkey milk is a safe and valuable source of nutrition. Further research is needed to better understand its health benefits and ensure the safety of milk produced in natural conditions. This research is important for expanding our knowledge of donkey milk, which could have positive implications for both animal farming and human health.

## 1. Introduction

Donkey milk is highly valued for its unique nutritional characteristics, particularly its similarity to human milk [1,2] and hypoallergenic properties [3], making it a suitable alternative for infants [4,5] and individuals of all ages with allergies to cow’s milk [6,7]. Understanding the nutritional composition of donkey milk is essential for evaluating its suitability for human consumption and its role in a balanced diet. In the future, the consumption of donkey milk is expected to increase globally and across Europe due to the rising prevalence of cow’s milk allergies. Prior studies have identified its rich content of essential nutrients, including proteins, ammino acids, fatty acids, lactose [8,9,10,11,12,13,14,15,16,17,18], and vital minerals such as calcium (Ca), phosphorus (P), sodium (Na), potassium (K), and magnesium (Mg) [13,19,20]. Trace elements like zinc (Zn), copper (Cu), and selenium (Se) also contribute to its nutritional profile [1,21,22]. Despite its nutritional advantages and recognized quality, the safety of donkey milk warrants detailed investigation due to the potential presence of various contaminants [23,24]. The presence of potentially toxic elements such as arsenic (As), lead (Pb), mercury (Hg), and cadmium (Cd) necessitates thorough safety assessments, as these contaminants pose significant health risks, especially to vulnerable populations [25,26,27,28]. Environmental contaminants, particularly heavy metals, are a public health concern due to their persistence, toxicity even at low concentrations, and accumulation in the food chain [29]. Chronic exposure to these elements, including Pb, Cd, Hg, and As, can lead to severe health issues [30,31].

Chemical contaminants, including heavy metals, can enter milk through contaminated feed and water, environmental pollution, or inadequate agricultural practices [32,33,34]. In addition to feed and environmental contamination, improper handling and storage of milk can further increase contaminant levels [35], posing risks to consumer health and potentially undermining the therapeutic potential of donkey milk.

The lactation period in donkeys varies between 180 and 350 days, with peak milk production typically occurring 4–5 weeks postpartum [11,36]. As a seasonal polyestrous species, donkeys can be managed to ensure year-round milk production [37,38]. Factors such as breed and seasonal changes significantly influence the milk’s composition [1,8,17,39]. The Balkan and Banat donkey breeds are particularly noteworthy for their adaptability and potential for high-quality milk production. The Balkan donkey, recognized as an indigenous breed in Serbia, has been the subject of studies focusing on microbiological quality and antimicrobial potential [40,41,42], while the Banat donkey, a robust and productive breed, has not yet been extensively studied [8,43]. These two breeds, reared in the Zasavica Natural Reserve, offer an excellent opportunity for investigating both nutritional value and safety.

Although studies have extensively examined heavy metal contamination in cow [44,45], goat [46], and sheep milk [47,48], research on their presence in donkey milk remains limited, especially in Serbia and neighboring regions. With the increasing popularity of organic and extensive farming systems, ensuring the safety and quality of milk produced in such systems has become critical.

This study aimed to comprehensively analyze the mineral content and safety of milk from Balkan and Banat donkey breeds across different seasons. It focused on determining the concentrations of essential minerals and trace elements (Ca, P, Na, K, Mg, Zn, Cu, Se), and potentially toxic elements (As, Pb, Hg, Cd). By bridging gaps in current knowledge, this research seeks to contribute valuable insights into the impact of environmental factors on the nutritional quality and safety of donkey milk.

Notwithstanding we acknowledge the importance of factors such as lactation period, parity, diet, and environmental conditions in influencing the mineral content of donkey milk. While our study primarily focuses on seasonal variations in essential minerals, trace elements, and potentially toxic elements in donkey milk from Banat and Balkan breeds in the Zasavica Nature Reserve, we understand that these additional factors may also play a role in shaping the results.

A key significance of our study is that it is the first of its kind on the mentioned donkey breeds, making a significant contribution to the expansion of knowledge as well as the preservation of indigenous breeds. Additionally, this is the first study to include a considerably large number of samples compared to previous research, and it covers a longer time period. It is also important to note that conducting research under extensive organic production conditions (free-range) is very challenging, which further enhances the value of our results.

## 2. Materials and Methods

### 2.1. Study Area

The study was conducted in the Zasavica Nature Reserve, located in the northern part of Serbia. The reserve covers a diverse landscape characterized by wetland ecosystems, including marshes, rivers, and floodplains, providing a natural habitat for wildlife, including the Banat and Balkan donkey breeds. The region is known for its rich biodiversity and ecologically significant environments. The climate of the Zasavica Nature Reserve is classified as temperate continental, with cold winters and warm summers, characterized by distinct seasonal variations. The average annual temperature in this area ranges from 10 °C to 14 °C, with temperatures dipping below freezing in winter and reaching above 30 °C during summer months. Precipitation is moderate, with rain predominantly occurring in the spring and autumn, which can influence the availability of nutrients in the local environment.

The donkey farm within the Zasavica Nature Reserve houses approximately 300 milking donkeys, making it the largest of such farms in Southeastern Europe. In addition to donkeys, the reserve is home to other animal species, including horses, Serbian Podolian cattle, Mangalitsa pigs, and various bird species. The donkeys are raised in an extensive production system, where they graze for nine months of the year and drink water from available springs within the reserve. The pasture consists of meadow grass, and the pastures are not fertilized as there is no need due to the high number of animals. During the winter months, the donkeys are kept indoors and are fed grain-based feed in the morning and the afternoon, primarily corn and triticale. They are fed together; no individual meals are provided. Ivermectin is used to treat parasites when the animals are brought inside and before they are returned to pasture. However, milking donkeys do not receive ivermectin due to its withdrawal period. The males in the herd are regularly rotated to prevent inbreeding. These males are sourced from small farms in the country, which may become problematic in the future as their numbers are decreasing. The core herd was formed by randomly purchasing individuals from across the country as well as neighboring countries (e.g., Bulgaria). After birth, the foals are weaned from suckling at 2.5–3 months of age but remain with their mothers to maintain lactation for an additional 2–3 months. If mastitis occurs, treatment is carried out with lard, and the donkeys are milked, but the milk is discarded and excluded from our research. Only milk from clinically healthy donkeys was sampled. Natural mating is practiced in the reserve, with one male present in each herd. Regarding milk production, the average yield per donkey is 300 mL, with a peak of 1000 mL during the height of lactation and 100 mL at the end of lactation. Some donkeys remain productive for 11–12 years. Breeding occurs from late May to early October since the animals are sensitive to cold, ensuring the foals are strong by winter. No breeding takes place from November to April. At any time, between 16 and 30 donkeys are milked.

### 2.2. Sampling

Raw donkey milk samples were collected throughout the year and during the entire duration of the project from the milk reservoirs, as 3 composed samples from all of the donkeys milked each month (9 pooled samples per season). Specifically, 3 composite samples were taken each month, meaning that every 3 months (i.e., 1 season), a total of 9 samples were collected. Over the course of the 4 seasons (12 months), which was the duration of the study, a total of 36 composite samples of donkey milk were collected. Milk sampling at the Zasavica Nature Reserve was carried out from July 2023 to June 2024. The samples were collected at regular monthly intervals, specifically during the first week of each month. Donkey milk was collected via tank sampling, and the specific animals milked each month were not always the same. However, we ensured that the samples were representative of the overall herd. Milk samples were taken from healthy donkeys of the Balkan and Banat breeds. The samples from both the Banat and Balkan donkey breeds were mixed to form a composite sample. This approach was used to represent the overall milk quality from the herd. It is also important to note that these breeds are not kept separately and are raised together in the reserve.

All samples were aseptically collected in containers made of food-safe materials suitable for future analyses. Specific protocols for collecting, transporting, and storing the samples were used. To prevent spoilage, all milk samples were immediately processed and stored under strict refrigeration conditions (at temperatures below 4 °C) following collection. The milk samples were transported to the laboratory in refrigerated containers (at a temperature of 4 °C). This ensured that the milk remained fresh until analysis. Additionally, milk samples were kept in sterile containers to prevent contamination, and the processing was carried out as soon as possible after collection to minimize any risk of degradation. Samples were stored in appropriate conditions in the laboratory according to standard operating procedures to maintain the integrity of the milk for chemical analysis. These measures ensured that spoilage was effectively prevented, and the milk samples remained suitable for accurate analysis throughout the study period.

### 2.3. Sample Preparation

Donkey milk samples were prepared for ICP-MS analysis using the acid digestion method in the Ethos system, Microwave Labstation (Milestone s.r.l., Sorisole, Italy).

In brief, 1 g of the donkey milk sample was mixed with 8 mL of diluted HNO_3_ acid (2 parts of 65% *w*/*w* acid and 1 part of deionized water) and 2 mL of H_2_O_2_ (30% *w*/*w*). The mixture was then digested at 180 °C and 40 bars for 30 min. After digestion, the resulting clear acid solutions were quantitatively transferred to volumetric flasks and diluted with deionized water to a final volume of 25 mL. The blank sample was prepared in the same manner, excluding the milk sample. All samples were prepared in duplicate.

### 2.4. Equipment and Analysis—Analysis of Contaminants and Mineral Elements in Donkey Milk

The elements of interest were determined using the Agilent ICP-MS 7700 (Agilent Technologies, Santa Clara, CA, USA), equipped with collision/reaction cell technology (Octopole Reaction System, ORS) to minimize spectral interferences.

### 2.5. Standard Preparation

Single-element standards of Ge, Rh, Lu, and Ir (all from CPI International, Amsterdam, The Netherlands) were used to prepare the internal standards. Calibration standards were prepared using single-element standards of Ca, P, Na, K, Mg, As, Pb, Hg, Cd, Zn, Cu, and Se (all from AccuTrace™ Reference Standards, New Haven, CT, USA). All standards were prepared daily.

### 2.6. Quality Control

For quality control, the reference material was treated and analyzed alongside the collected donkey milk samples. A certified milk sample, Test Material 07293 “Metallic Contaminants, Powder Milk” (Fapas, Fera Science Ltd., Sand Hutton, UK), was analyzed. Since the reference sample did not provide values for some elements, it was spiked with known concentrations of Ca, P, Na, K, and Mg (0.5 mg/g), and Zn, Cu, and Se (0.1 mg/g). Recoveries for the analyzed elements ranged from 95% to 110%.

### 2.7. Calculations and Statistical Analysis

The limit of detection (LOD) was calculated as three times the standard deviation (SD) of the element concentration in the calibration blanks, while the limit of quantification (LOQ) was calculated using the formula: LOD = 0.3 × LOQ. The correlation coefficient for all calibrations was R > 0.95.

Data were analyzed using Excel (Microsoft Office 2013) with the Data Analysis add-on. Various statistical tests, including ANOVA (single factor) and the F-test, were applied to assess the results. Results are presented as mean values ± standard deviation, with a significance level of *p* < 0.05 used to determine statistically significant differences between means.

In cases where the obtained values were below the LOD, we applied a replacement of that value with a specific value, i.e., the values below the detection limit were replaced by half of the limit of detection (LOD) values of the instrument for statistical analysis.

## 3. Results

### 3.1. Essential Minerals

The results of the analysis of pooled donkey milk samples are presented in Table 1. The concentration of Ca throughout the year ranged from 500 to 900 mg/kg, with the highest average value observed during the autumn period (744.44 ± 52.70 mg/kg). The concentration of P ranged from 200 to 500 mg/kg, and the highest average value was found in the milk sampled in the spring, which was also the case for the concentrations of Na, K, and Mg. The analysis of variance indicates that the values between the groups defined by the seasons did not show statistically significant differences for certain examined minerals (Na and Mg), since the obtained values for the F-test were lower than the F-critical values. Statistically significant differences were recorded in the measured values of Ca, P, K, as well as in the Ca/P ratio between the formed groups. The F-test values were significantly higher than the F-critical values (Table 1).

### 3.2. Potentially Toxic Elements

Arsenic (As) concentrations ranged from <LOD to 0.004 mg/kg, measured in milk samples collected during the autumn and winter months. In the autumn samples, six pooled samples had values below 0.001 mg/kg, as was the case with the winter samples, where the mean value was 0.001 mg/kg. In the spring, eight of nine pooled samples had concentrations below 0.001 mg/kg, and one sample had a concentration of 0.001 mg/kg. The variance analysis of the values between the groups defined by the seasons showed statistically significant differences in the concentration of As across different seasons, with the obtained F value significantly higher than the F-critical value.

In the spring months (April, May, June), two pooled samples taken in April contained lead (Pb) levels above the permissible limit (0.032 and 0.041 mg/kg). A third pooled sample from April had a Pb concentration at the detection limit, 0.001 mg/kg. In all three pooled samples from May, the Pb concentration was below the detection limit (<0.001 mg/kg), as well as in one sample from June, while in the other two pooled samples from June, the Pb levels were 0.004 and 0.005 mg/kg. In six of nine pooled samples from the summer months, the Pb concentration was below the detection limit (<0.001 mg/kg). In the autumn sampling, Pb levels were below the detection limit in five pooled samples, while in the winter months, seven pooled samples had Pb concentrations below the detection limit. Mercury (Hg) levels were below the detection limit (<0.001 mg/kg) in five pooled samples from spring, three samples had a concentration of 0.001 mg/kg, and one had 0.007 mg/kg. In the winter period, Hg was below the detection limit in eight pooled samples and equal to 0.001 mg/kg in one sample. In autumn, Hg was below the detection limit in six pooled samples, and in two samples, it was equal to that value. In the summer period, Hg was below the detection limit in five pooled samples. Cadmium (Cd) levels were below the detection limit (<0.001 mg/kg) in six samples from spring, eight from summer, and all nine pooled samples from autumn and winter. No statistically significant differences were found between the concentrations of Pb, Hg, and Cd in the donkey milk during different seasons (F < Fcrit).

### 3.3. Trace Elements

When it comes to trace elements, the variance analysis revealed significant variation in the concentration of Cu in milk during different seasons (Table 1), while no statistically significant variation was found for the measured concentrations of Zn and Se in the donkey milk samples, and the F values were lower than the F-critical value.

## 4. Discussion

In our study, we found high levels of Ca and Se across all four seasons (Table 1). Previous research has also established that this is an important characteristic of donkey milk [1]. The previous results [13,49] showed that donkey milk is a good source of Ca, P, Na, K, Mg, Zn, and Cu, which was also confirmed in our research. The climatic and environmental factors of the Zasavica are important, as they can directly affect the forage quality and mineral composition of the pasture, which in turn may influence the mineral content of donkey milk. Seasonal changes in temperature and rainfall can alter the nutrient profile of the plants available to the donkeys, thereby impacting the concentrations of essential minerals in their milk. The results of previous studies [7,12,13,17,18,19,20,50,51] and our own research regarding minerals and trace elements in donkey milk and in human, cow, sheep, goat, and mare milk [52] are summarized in Table 2. Donkey milk is more suitable for human consumption compared to cow milk when considering the Ca/P ratio [19]. The levels of Ca, P, Na, and Mg in donkey milk are lower in both our study and previous studies compared to these elements in the milk of other domestic animals, such as cows, sheep, and goats [52]. The content of Na, K, and Cu significantly differs in donkey milk compared to mare milk [20,52], while the levels of Ca, Se, and Zn are similar [20,52]. Analysis of donkey milk revealed that Zn is the most abundant trace element, and its concentration is significantly higher than in human milk, which is also an important nutritional characteristic of donkey milk [1,8,17,21]. This is particularly relevant when considering the role of Zn in growth and immune system development during childhood and adolescence. In mare milk, a higher concentration of Cu was found compared to donkey milk [13], while Cu levels in donkey milk are about two times higher than in human and cow milk, and significantly higher than in sheep and goat milk. The Se content in donkey milk is about seven times higher than in human milk and ten times higher than in cow and sheep milk.

The analysis of donkey milk from the Banat and Balkan breeds in the Zasavica Nature Reserve reveals varying levels of essential minerals and trace elements, which are crucial for meeting the nutritional requirements of different age groups. For infants and young children, donkey milk can be a valuable source of calcium, phosphorus, and magnesium, which are essential for bone development and growth. However, while it provides adequate amounts of certain nutrients, it may not fully meet the recommended daily intake for all essential elements, particularly for older children and adults who require higher amounts of some minerals. Therefore, while donkey milk could contribute to the overall nutritional needs, it should be considered as part of a balanced diet rather than a sole nutrient source. This highlights the need for further studies to assess the adequacy of donkey milk for different age groups and ensure its suitability and safety for human consumption.

The presence of toxic elements such as lead in donkey milk may be influenced by various environmental factors within the Zasavica Nature Reserve. One potential source of these contaminants is the local soil composition. The surrounding area is known to have a history of industrial activity and agricultural use, which could lead to soil contamination by heavy metals. These metals can accumulate in the soil through long-term exposure to industrial runoff, fertilizers, or pesticides, and can subsequently be taken up by plants, which donkeys may consume as forage. Additionally, water sources in the region, such as rivers and lakes, could contribute to the presence of toxic elements. Pollution from agricultural runoff or nearby industrial waste can contaminate water supplies, allowing harmful substances to enter the food chain. In particular, seasonal flooding events could exacerbate the spread of contaminants, as water may transport heavy metals from soil and industrial sites to grazing areas. Furthermore, traffic, transportation, environmental pollution, and climate change may alter the natural processes of contamination, leading to increased levels of toxic elements in the forage, which could be absorbed by the donkeys and detected in their milk. Therefore, understanding the sources and pathways of contamination in the local environment is crucial for assessing the potential risks of toxic element exposure in donkey milk produced in the Zasavica Nature Reserve.

The contamination of donkey milk with heavy metals is of particular importance due to its unique group of consumers who are drawn to this milk for its hypoallergenic properties and nutritional benefits [23]. Similar to other animals, the bioaccumulation of heavy metals in donkey milk occurs through various exposure pathways, including contaminated feed, water, and environmental exposure [22,53]. Heavy metals are naturally present in soil, water, and air or result from anthropogenic activities such as industrial pollution, agricultural practices, and urbanization [32,33,34,54]. Although the research data on heavy metal contamination in donkey milk are limited, it can be inferred that donkeys, like other grazing animals, may accumulate these elements if they graze on contaminated pastures or consume polluted water. Contaminated feed is also considered a potential source of heavy metals in milk [55]. Previous studies have documented the bioaccumulation of heavy metals in the milk of various animals, primarily cows [56,57]. However, it is important to note that donkeys are monogastric animals, unlike cows, which are ruminants. This fundamental difference influences the transport of toxic substances from feed to milk, as the digestive processes in monogastric animals differ from those in ruminants [23]. While specific studies on the pathways of heavy metal bioaccumulation in donkeys are scarce, it is hypothesized that contaminated feed and water are primary sources. Additionally, environmental exposure and contaminated equipment may contribute to the presence of contaminants in donkey milk [22].

In our study, we observed that certain donkey milk samples exceeded the permitted limit for lead concentrations, raising concerns about potential health risks associated with its consumption. Lead is a neurotoxin that can cause significant harm, particularly in vulnerable populations such as infants, young children, and pregnant women. Chronic exposure to lead can result in developmental delays, cognitive impairments, and other neurological disorders, which is especially concerning given the higher absorption rates in younger individuals [25,29,30,31,32]. As donkey milk is increasingly consumed as an alternative to cow’s milk, it is crucial to monitor and regulate lead levels to protect public health, especially in at-risk age groups. The need for future research to better understand the physiological processes in donkeys is evident. The limited literature on heavy metal contamination in donkey milk highlights the importance of expanding this field of study. Existing research from Turkey, Romania, and Italy [22,53,58,59] has shown that, in most cases, heavy metal residues in donkey milk are below harmful levels for human health. However, occasional exceedances of permissible levels for lead and cadmium have been reported, particularly in studies from Italy, where 11% of milk samples surpassed the European lead limit of 0.02 mg/kg [60] which is also the case in our study (Table 1). Namely, two pooled samples taken in April contained lead levels above the permissible limit (0.032 and 0.041 mg/kg). The presence of lead in milk may stem from various sources, including feed grown near roads, exposure to polluted environments, aging, and poorly maintained water supply systems, or pesticide and fertilizer use. Seasonal fluctuations in metal concentrations in milk and animal feed have been observed, suggesting that environmental conditions play a role in these variations [22,23].

There are no data on the consumption of donkey milk per capita in Serbia. What is known is that the number of donkeys in the country is small, and donkey milk is consumed sporadically, both due to its high price and limited availability. Because of this, it is very difficult to conduct a risk analysis when it comes to potentially toxic elements, as well as to determine to what extent this food item meets the needs for mineral substances. All of these facts open opportunities for future research and for the popularization of donkey milk.

Overall, this study underscores the importance of monitoring contaminants and residues in donkey milk, considering the growing demand for this milk due to its unique health benefits. Further research on seasonal and environmental factors influencing contamination levels, as well as physiological mechanisms specific to donkeys, is crucial for ensuring the safety and quality of donkey milk.

## 5. Conclusions

Our study highlights the seasonal variation in the mineral and trace element content of donkey milk from the Banat and Balkan breeds. The milk of these breeds is a good source of essential minerals, although the presence of heavy metals and other potentially toxic elements poses a potential food safety risk. Despite very low contamination levels, primarily below legally permitted limits, continuous monitoring is essential, especially considering the exposure of vulnerable populations, such as children, to trace amounts of these substances. Given the growing demand for donkey milk, particularly in response to rising cow’s milk allergies, further research should focus on risk assessment and the development of preventive measures. Our study contributes valuable knowledge, especially as it is the first to investigate these donkey breeds under organic farming conditions over an extended period and with a larger sample size compared to previous research. Future research should focus on larger-scale studies, risk assessment, and the development of innovative preventive measures, building on the existing knowledge of chemical contamination in donkey milk. Such efforts will be essential to ensure the safety and quality of this increasingly popular alternative milk source.

## Figures and Tables

**Table 1:** Essential minerals (mg/kg), heavy metals (mg/kg), and trace elements (mg/kg) in donkey milk composite samples through four seasons.

Elements (Unit)	Spring		Summer		Autumn		Winter		ANOVA	
	Range	Mean Value ± SD	Range	Mean Value ± SD	Range	Mean Value ± SD	Range	Mean Value ± SD	*p*-Value	F
Ca ^1^ (mg/kg)	500–800	655.56 ± 113.04	400–900	588.89 ± 153.66	700–800	744.44 ± 52.70	600–800	733.33 ± 70.71	<0.05	4.32
P ^2^ (mg/kg)	500–600	533.33 ± 50	200–500	388.89 ± 78.17	300–500	355.56 ± 72.65	300–600	411.11 ± 126.93	<0.05	7.21
Na ^3^ (mg/kg)	400–500	444.44 ± 52.70	200–800	388.89 ± 169.15	200–500	355.56 ± 88.19	300–500	355.56 ± 72.65	0.25	1.43
K ^4^ (mg/kg)	500–800	711.11 ± 105.41	200–800	444.44 ± 235.11	500–700	566.67 ± 70.71	400–700	500 ± 100	<0.05	5.86
Mg ^5^ (mg/kg)	50–100	84.44 ± 18.10	30–100	71.11 ± 34.44	40–100	81.11 ± 21.47	40–100	75.56 ± 22.42	0.68	0.51
As ^6^ (mg/kg)	<0.001–0.001	0.0006 ± 0.0002	<0.001–0.01	0.0033 ± 0.0029	<0.001–0.004	0.0013 ± 0.0013	<0.001–0.004	0.0011 ± 0.0012	<0.05	4.49
Pb ^7^ (mg/kg)	<0.001–0.041	0.009 ± 0.016	<0.001–0.013	0.004 ± 0.005	<0.001–0.007	0.002 ± 0.002	<0.001–0.004	0.001 ± 0.001	0.16	1.82
Hg ^8^ (mg/kg)	<0.001–0.007	0.0014 ± 0.0021	<0.001–0.006	0.0014 ± 0.0018	<0.001–0.001	0.0006 ± 0.0002	<0.001–0.001	0.0006 ± 0.0002	0.41	1.00
Cd ^9^ (mg/kg)	<0.001–0.006	0.0014 ± 0.0018	<0.001–0.001	0.0006 ± 0.0002	<0.001	0.0005	<0.001	0.0005	0.10	2.30
Zn ^10^ (mg/kg)	1.45–3.46	2.19 ± 0.75	1.33–3.49	2.21 ± 0.61	1.44–2.67	2.06 ± 0.38	1.33–2.48	2.08 ± 0.37	0.92	0.17
Cu ^11^ (mg/kg)	0.12–0.53	0.31 ± 0.14	0.41–0.98	0.68 ± 0.22	0.22–0.55	0.35 ± 0.10	0.26–0.41	0.34 ± 0.05	<0.05	14.43
Se ^12^ (mg/kg)	0.01–0.03	0.02 ± 0.01	0.01–0.05	0.03 ± 0.01	0.02–0.22	0.05 ± 0.07	0.01–0.03	0.02 ± 0.01	0.26	1.40
Ca/ P ratio	1.00–1.33	1.22 ± 0.11	1.00–3.50	1.63 ± 0.79	1.40–2.67	2.17 ± 0.47	1.17–2.67	1.93 ± 0.59	<0.05	4.98

Notes: ^1^ Ca—calcium; ^2^ P—phosphorus; ^3^ Na—sodium; ^4^ K—potassium; ^5^ Mg—magnesium; ^6^ As—arsenic; ^7^ Pb—lead; ^8^ Hg—mercury; ^9^ Cd—cadmium; ^10^ Zn—zinc; ^11^ Cu—copper; ^12^ Se—selenium; Detection limits (mg/kg) of As, Cd, Hg, and Pb = 0.001 mg/kg; Cu = 0.006 mg/kg; Zn = 0.007 mg/kg; The results are presented as the range, mean value, standard deviation (SD) (n = 9); *F crit* = 2.90.

**Table 2:** The results of previous studies [7,12,13,17,18,19,20,50,51] and our own research regarding minerals and trace elements in donkey milk and in human, cow, sheep, goat, and horse milk [52].

Elements mg/kg	Previous Studies [7,12,13,17,18,19,20,50,51]	Our Results	Human Milk [52]	Cow Milk [52]	Sheep Milk [52]	Goat Milk [52]	Horse Milk [52]
Ca ^1^	543.6–689	588.9–744.4	271.84–330.10	1087.38–1194.17	1543.69–2349.51	825.24–1922.33	485.44–1310.68
P ^2^	410–518	355.6–533.3	135.92–417.48	572.82–1155.34	1203.88–1699.03	766.99–1485.44	194.17–1174.76
Mg ^3^	61.3–88.9	71.1–84.4	29.13–38.83	67.96–116.50	155.34–242.72	97.09–349.51	29.13–116.50
K ^4^	657–1102.7	444.4–711.1	514.56–601.94	1029.13–1582.52	912.62–1572.85	1359.22–2349.51	242.72–844.66
Na ^5^	370–1730	355.6–444.4	97.09–174.76	563.11	291.26–728.16	271.84–572.82	77.67–563.11
Zn ^6^	0.50–2.24	2.06–2.21	1.94–3.88	2.91–5.34	3.88–8.74	3.88–5.83	0.87–6.21
Cu ^7^	0.151–0.310	0.31–0.68	0.19–0.58	0.10–0.78	0.29–0.49	0.19–0.49	0.19–1.07
Se ^8^	0.151	0.02–0.05					

Notes: ^1^ Ca—calcium; ^2^ P—phosphorus; ^3^ Mg—magnesium; ^4^ K—potassium; ^5^ Na—sodium; ^6^ Zn—zinc; ^7^ Cu—copper; ^8^ Se—selenium.

## Data Availability

Data are contained within the article.
